# Frequency of narcissistic personality disorder in a counseling center population in China

**DOI:** 10.1186/s12888-019-2185-5

**Published:** 2019-07-05

**Authors:** XueFeng Jiang, JunJie Wang, Wei Sun, LiHua Xu, XiaoChen Tang, HuiRu Cui, YanYan Wei, Li Hui, Yi Qiao, JiJun Wang, TianHong Zhang

**Affiliations:** 1 Shanghai Mental Health Center, Shanghai Jiaotong University School of Medicine, Shanghai Key Laboratory of Psychotic Disorders (No.13dz2260500), 600 Wanping Nan Road, Shanghai, 200030 People’s Republic of China; 2 Institute of Mental Health, Suzhou Guangji Hospital, The Affiliated Guangji Hospital of Soochow University, Soochow University, Suzhou, 215137 Jiangsu China; 3 Department of Neurosurgery, Pu Nan Hospital, Shanghai, 200125 China; 4 CAS Center for Excellence in Brain Science and Intelligence Technology (CEBSIT), Chinese Academy of Science, Shanghai, People’s Republic of China; 5 Bio-X Institutes, Key Laboratory for the Genetics of Developmental and Neuropsychiatric Disorders (Ministry of Education), 600 Wanping Nan Road, Shanghai, 200030 People’s Republic of China

**Keywords:** Narcissism, DSM, Diagnosis, Narcissistic personality disorder, China

## Abstract

**Background:**

Narcissistic personality disorder (NPD) has never been applied in Chinese clinical practice, and the distribution of NPD in the clinical population of China is largely unknown. The current study uses two-stage clinic-based screening to investigate the frequency and clinical features of NPD in a Chinese help-seeking sample.

**Methods:**

A total of 1402 consecutive outpatients ages 18–60 were recruited during their visit to the Shanghai Mental Health Center and screened with the Personality Diagnostic Questionnaire Fourth Edition Plus (PDQ-4+) and Structured Clinical Interview for the Diagnostic and Statistical Manual of Mental Disorders (DSM-IV) Axis II (SCID-II). The structured clinical interview was administered to estimate the rate of NPD and the frequency of each disorder criterion.

**Results:**

The frequency estimate of NPD in the total sample was 4.0%. Among the 56 outpatients who met the criteria for NPD, there were more males than females, and many had a better educational background. The SCID-II interviews revealed high frequencies of diagnostic criterion 1 ("exaggerated sense of self-importance. NPD likely overlaps with Histrionic PD, Borderline PD, and Paranoid PD. This two stage screening method can enhance detection of Chinese NPD patients in clinical settings.

**Conclusions:**

Narcissism pathology is not rare in the Chinese psychiatric community when using the DSM-IV NPD criteria. Existing evidence suggests, at least indirectly, that there are important benefits of NPD diagnosis in psychiatric practice.

## Background

Narcissistic Personality Disorder (NPD) was first recognized in the Diagnostic and Statistical Manual of Mental Disorders, third edition (DSM-III) [1] in 1980. NPD patients are featured as having an exaggerated (fantastic or behavioral) behavior pattern requiring praise and lacking empathy. The disorder is widely used in clinical practice and widely accepted as an abnormal personality characteristic in North America [2, 3]. In the Chinese Classification and Diagnosis of Mental Diseases, Third Edition (CCMD-3) [4] in 2001, NPD was classified as a category of other or unspecific personality disorders [F60.8-F60.9]. Although NPD has not been included in the 11th revision of the International Classifications of Diseases and Related Health Problems (ICD-11), a large number of studies in North America in the past 30 years have demonstrated the reliability of this diagnostic validity and its independence as a diagnostic category. Therefore, the diagnostic criteria for NPD have been retained from the DSM-III to the DSM-5 [5] and have been revised to varying degrees. Notably, the “alternative model” described in the DSM-5 has curtailed the PD categories to six specific PDs [6], which include NPD.

Although the clinical features of NPD are distinct, the distribution of NPD in the Chinese clinical population remains largely unknown due to lack of appropriate diagnostic classification in Chinese clinical practice [7]. Moreover, the high rates of overlap between NPD and other types of PDs found in previous studies [3, 8], make it more difficult to generalize NPD in China [9]. However, narcissism is a very important issue in the theoretical study of psychology and psychotherapy. In clinical practice, effective identification of NPD is not only important for the diagnosis of mental disorders and differential diagnosis, but is also directly related to the formulation of treatment strategies. To be specific, affective relevant symptoms in NPD patients may be easier identified and this makes the antidepressant effects become the only target of treatment. We speculate that make a diagnosis of NPD may represent a new target for the development of effective therapeutic strategies (such as psychoanalysis [10]) to guide systematic analysis of the grandiose self. This diagnosis process also leads to a shift from symptom-centered treatment to personality rehabilitation, by the gradual integration of a grandiose, split-off self into a more integrated stable concept of the self. [11] This study is an exploratory study on the preliminary clinical application of NPD in China. The distribution of NPD, clinical and demographic characteristics, will be investigated in order to discuss the feasibility of the application of NPD diagnostic criteria in China.

## Methods

### Subjects and procedures

This study was approved by the Research Ethics Committee of the Shanghai Mental Health Center (SMHC) and conducted following the tenets of the Helsinki Declaration. Every 10th outpatient in the psychocounseling clinics was selected and a total of 1402 consecutive outpatients were recruited. All the participants gave written informed consent at the recruitment stage of the study. Study inclusion criteria were: between 18 and 60 years of age and with an educational background of at least junior middle school. Exclusion criteria were: outpatients with acute attacks of psychoses, severe somatic diseases, and diagnoses of mental retardation or dementia. Details of the study procedures can be found elsewhere [7, 12–15].

Outpatients were screened using the Personality Diagnostic Questionnaire Fourth Edition Plus (PDQ-4+) [16]. Positive screening was defined as a total score of 29 or higher on the PDQ-4+, or specific PD subscale scores of > 4 or 5. Outpatients whose PDQ-4+ test results were positive referred to two senior psychiatrists (5 years’ experience of psychiatry), each of whom received 2 weeks of training to perform the Structured Clinical Interview for DSM-IV Axis II (SCID-II). NPD diagnosis was confirmed using the questionnaire and face-to-face interview with a two-stage process.

### Measures

PDs were assessed with the PDQ-4+ and SCID-II. Our previous study [7] and other research has validated that the PDQ-4+ has a high sensitivity (0.89) and moderate specificity (0.65) for PD screening. The SCID-II [17] was designed to assess the DSM-IV 10 PDs. Our team translated and implemented the Chinese version of the SCID-II. Besides, previous reports have demonstrated that the Chinese version SCID-II has a median coefficient for internal consistency of 0.70, with a relatively high test–retest reliability (0.70). In the DSM-IV, there are nine diagnostic criteria for NPD. The PDQ-4+ and SCID-II were derived from DSM-IV and have a one-to-one relationship with it. Therefore, they can better reflect the diagnostic criteria for NPD in the DSM-IV. The corresponding relationship between the PDQ-4+, SCID-II, and the DSM-IV criteria can be found in Table 1.

**Table 1:** Correspondence between DSM-IV diagnostic criteria for narcissistic personality disorder (NPD) in PDQ-4+ and SCID-II

Diagnostic Criteria (DSM-IV, NPD)	Screen	Interview
	PDQ^4+^(NPD)	SCID-II (NPD)
A long-term pattern of abnormal behavior characterized by exaggerated feelings of self-importance, an excessive need for admiration, and a lack of empathy. The disorder begins by early adulthood and is indicated by at least five of the following:		
(1) An exaggerated sense of self-importance (e.g., exaggerates achievements and talents, expects to be recognized as superior without commensurate achievements)	I have achieved much more than others think. (Item-5)	Most people do not appreciate your extraordinary talents or achievements. (Item-73).
		Some people say that you have too high self-evaluation. (Item-74).
(2) Preoccupation with fantasies of unlimited success, power, brilliance, beauty, or ideal love	I often find myself thinking about what an important person I am or how I am going to be. (Item-18)	You say you often think that 1 day you will gain power, reputation or praise. (Item-75).
		You say you often think that 1 day you will have a perfect love story. (Item-76).
(3) Believes he is “special” and can only be understood by, or should associate with, other special or high-status people (or institutions)	Only some special person can really appreciate and understand me. (Item-31).	When you encounter problems, you almost always insist on meeting senior leaders. (Item-77).
		You think it’s only worth spending time with special or important people. (Item-78).
(4) Requires excessive admiration	I need people to pay attention to me or to praise me. (Item-44).	People pay attention to or envy you in some way, which is very important to you. (Item-79).
(5) Has a sense of entitlement	When a salesperson makes me wait in front of the counter or wait for a long time, I tend to feel indignant and angry. (Item-57)	When certain rules or social conventions interfere with you, you do not think it is necessary to comply with them. (Item-80).
		You often feel that others are giving you special treatment. (Item-81)
(6) Selfishly takes advantage of others to achieve his own ends	Some people think I use others. (Item-68).	You often feel that it is necessary to offend some people in order to get what you want. (Item-82).
		You often have to put your needs above others. (Item-83).
		You often want others to look at your face and do what you want without doubt. (Item-84).
(7) Lacks empathy	People often blame me for not realizing that they are in a bad mood. (Item-73).	You often think that it doesn’t matter how to treat others’ thoughts or feelings. (Item-85).
(8) Is often envious of others or believes that others are envious of him	Some people are jealous of me. (Item-79).	When others do well, you will be angry. (Item-86).
		You feel that people are often jealous of you. (Item-87).
(9) Shows arrogant, haughty, patronizing, or contemptuous behaviors or attitudes	Others think I’m arrogant. (Item-92).	You think that few people are worth your time or attention. (Item-88).

Note: PDQ-4+: The Personality Diagnostic Questionnaire Version 4 plus. SCID-II: Structured Clinical Interview for DSM-IV Axis II

### Statistical analysis

To explore whether demographic variables were risk factors for the NPD group, the frequency rates and odds ratios (OR) were examined, and 95% confidence intervals (CIs) were estimated. Group comparisons of demographic and clinical variables were evaluated using Pearson Chin-Square tests for categorical variables and two-tailed tests for continuous variables. Significance was reached when *p* < 0.05. We presented the positive rate of each NPD diagnostic criteria (DSM-IV) identified by the PDQ-4+ and SCID-II. Finally, binary logistic regression analysis was used to explore independent predictive factors for the outpatients who were diagnosed or comorbid with DSM-IV NPD and calculate OR with *p*-values and 95% CIs.

## Results

In this study, 1511 outpatients were randomly selected from the psychological counseling department. Among them, 1402 (92.8%) were willing to complete the study. Among the 1402 subjects, the proportion of women (*n* = 761, 54.3%) was slightly higher than that of men (*n* = 641, 45.7%). The average age was 30.5 ± 9.6 years old. There were 458 (32.7%) aged 18–24 years old, 533 (38.0%) aged 25–34 years, 263 (18.8%) aged 35–44 years, and the remaining 148 people (10.5%) were aged over 45 years old. The majority sample of the current study was younger than 35 years old (70.7%). There was no significant difference in age between the male and female groups (*t* = 1.55, *p* > 0.05). There were 986 (70.3%) screened positive for PD by the PDQ-4+. A total of 539 (38.4%) patients were diagnosed with PD. The frequency of PD was 38.4% (95% CI, 35.9–40.9%). In addition, the patients with PD were younger, more likely to have single marital statuses, and had fewer years of education than those without PD.

There were 56 patients who met the criteria for NPD, with a frequency rate of 4.0% (Table 2). The frequency of NPD was higher in males than in females (OR = 4.9, 95% CI: 2.5–9.3), by about a factor of five. NPD patients completed more years of education (OR = 0.4, 95% CI: 0.2–0.8) than other patients.

**Table 2:** Prevalence and demographics of narcissistic personality disorder (NPD) in Chinese psychological counseling outpatients

	In total	NPD(n)	(%)	95% *CI*	Comparison	*OR*	95% *CI*
Sample in all	1402	56	4.0	2.97%~ 5.03%	–	–	–
Gender							
1. Male	641	45	7.0	5.04%~ 9.00%	1~2	4.857	2.533~9.311
2. Female	761	11	1.4	0.60%~ 2.29%			
Age							
1. 18–28(years)	730	30	4.1	2.67%~ 5.55%	1~2	1.062	0.635~1.777
2. 29–60(years)	672	26	3.9	2.41%~ 5.33%			
Educational background							
1. High school degree or below	596	13	2.2	1.01%~ 3.35%	1~2	0.409	0.222~0.753
2. College degree or above	806	43	5.3	3.78%~ 6.89%			
Personal income (/ month / RMB)							
1. ~ 1000	518	22	4.2	2.51%~ 5.98%	1~2	1.499	0.776~2.896
2. 1000~3000	494	14	2.8	1.37%~ 4.30%	1~3	0.828	0.459~1.496
3. 3000~	390	20	5.1	2.94%~ 7.32%	2~3	0.553	0.283~1.080

Note: *95% CI* 95% confidence interval, *OR* odds ratio

The positive rate of each diagnostic criterion of NPD in the self-report questionnaire and structured interview in the overall sample is listed in Table 3. The diagnostic criterion 4 (requiring excessive envy) had the highest positive rate in both the self-report and interview, followed by the self-rated subjects who considered themselves “special” and empowered (criterion 3, 5). In addition, in gender comparisons, except for criteria 4 and 9, there were more males than females who reported NPD symptoms, especially for criterion 1(an exaggerated sense of self-importance).

**Table 3:** Positive rate of each NPD diagnostic criteria (DSM-IV) identified by PDQ-4+ and SCID-II, and their sex difference

Diagnostic Criteria	Screened Positively by PDQ^4+^(*N* = 1402 in total)			Interviewed Positively by SCID-II (*N* = 986 in total)							
				In total PD sample		Male (*N* = 460)		Female (*N* = 526)		Male vs. Female comparison	
	n	Positive rate(%)	95% CI	n	Positive rate(%)	n	Positive rate(%)	n	Positive rate(%)	*Χ* ^*2*^	*P*
1	552	39.4	34.6–44.3	164	16.6	104	22.6	60	11.4	22.207	0.000***
2	612	43.7	38.8–48.6	223	22.6	130	28.3	93	17.7	15.696	0.000***
3	647	46.1	41.2–51.1	98	9.9	64	13.9	34	6.5	15.212	0.000***
4	762	54.4	49.5–59.3	233	23.6	117	25.4	116	22.1	1.555	0.212
5	634	45.2	40.3–50.1	180	18.3	100	21.7	80	15.2	7.012	0.008**
6	239	17.0	13.3–20.7	224	22.7	119	25.9	105	20.0	4.878	0.027*
7	278	19.8	15.9–23.8	79	8.0	48	10.4	31	5.9	6.867	0.009**
8	520	37.1	32.3–41.9	97	9.8	55	12.0	42	8.0	4.364	0.037**
9	428	30.5	25.9–35.1	117	11.9	63	13.7	54	10.3	2.760	0.097

To investigate the specificity of the NPD diagnostic criteria, we explored the positive rates of NPD criteria in other types of PDs (Table 4). NPD is basically different from other types of PDs (positive rate is higher than other types), but some diagnostic items have high positive rates in other PDs. For example, for criterion 4 (needs too much envy), the positive rate in Histrionic PD is as high as 60.9%, which is close to 69.6% of NPD’s positive rate. In addition, according to the positive rate of NPD patients, the importance of self-exaggeration (Criterion 1) is the highest common symptom, followed by Criterion 2 (73.2%).

**Table 4:** Positive rate (%) of each NPD diagnostic criteria (DSM-IV) among specific PD

Diagnostic Criteria	NPD	HIS	BOR	ANT	PAR	SCH	SCHT	AVO	DEP	OBC	DPS	PAS
	*n* = 56	n = 46	n = 98	*n* = 16	*n* = 71	*n* = 66	*n* = 74	*n* = 146	*n* = 40	*n* = 121	*n* = 72	*n* = 57
1	82.1	26.1	16.3	31.3	32.4	12.1	27.0	10.3	2.5	26.4	5.6	35.1
2	73.2	37.0	34.7	12.5	39.4	15.2	37.8	21.9	17.5	29.8	20.8	28.1
3	58.9	15.2	14.3	18.8	22.5	4.5	16.2	6.2	2.5	16.5	4.2	12.3
4	69.6	60.9	29.6	25.0	29.6	7.6	29.7	15.8	27.5	31.4	16.7	33.3
5	66.1	15.2	23.5	25.0	33.8	24.2	23.0	14.4	15.0	23.1	13.9	36.8
6	67.9	30.4	37.8	25.0	40.8	21.2	33.8	19.2	25.0	25.6	29.2	42.1
7	50.0	6.5	9.2	25.0	12.7	18.2	8.1	8.9	7.5	11.6	5.6	21.1
8	58.9	19.6	16.3	18.8	18.3	7.6	17.6	8.2	7.5	22.3	6.9	17.5
9	46.4	13.0	15.3	12.5	18.3	21.2	17.6	11.6	7.5	15.7	13.9	15.8

Note: Cluster A PD: Paranoid PD (PAR); Schizoid PD (SCH); Schizotypal PD (SCHT); Cluster B PD: Histrionic PD (HIS); Narcissistic PD (NAR); Borderline PD (BOR); Antisocial PD (ANT); Cluster C PD: Avoidant PD (AVO); Dependent PD (DEP); Obsessive–compulsive PD (OBC); In the appendix of DSM-IV: Depressive PD (DPS);Passive-aggressive PD (PAS)

To identify risk factors for NPD diagnoses, logistic regression (forward stepwise) analyses were performed. The presence of NPD was applied as the dependent variable, while demographic characteristics and other PD diagnoses were listed as independent variables. As shown in Table 5, demographic factors, such as male gender, an older age, and a higher educational level, were associated with the diagnosis of NPD. Patients having other PDs, such as Histrionic PD, Borderline PD, Paranoid PD, and Schizotypal PD, was a significant predictor of meeting criteria for NPD diagnosis. Those patients with Avoidant PD were less likely to be diagnosed with NPD. The strongest effect was derived from gender.

**Table 5:** Logistic regression for risk factors predicting the diagnosis of Narcissistic PD

Variable	Beta	SE	Wald statistic	*P* value	Odds ratio	95%CI	
Gender	2.774	0.467	35.298	0.000	16.025	6.417	1.106
Age (years)	0.065	0.018	12.840	0.000	1.067	1.030	40.016
Educational Level	1.541	0.386	15.897	0.000	4.669	2.189	9.960
Paranoid PD	1.390	0.440	9.987	0.002	4.016	1.696	9.511
Schizotypal PD	1.107	0.542	4.175	0.041	3.025	1.046	8.744
Histrionic PD	2.233	0.539	17.131	0.000	9.326	3.240	26.845
Borderline PD	1.727	0.504	11.729	0.001	5.625	2.093	15.115
Avoidant PD	−2.473	1.053	5.514	0.019	0.084	0.011	0.664

## Discussion

The diagnostic criteria for NPD have been changing and developing since NPD was officially listed in the DSM-III in 1980. At the same time, the debate over the validity and reliability of NPD has never stopped [6, 18]. Because of these controversies, NPD has rarely been used in clinical practice of psychiatry in China, and therefore the frequency of NPD in the Chinese clinical population is largely unknown. Otherwise, it has been reported that the frequency rate of NPD has been increasing recently in other countries [8, 19, 20]. To our knowledge, our research group is the first to investigate the distribution of the NPD outpatients in an epidemiological way in a clinical setting in mainland China. The frequency of NPD in an outpatient counseling setting was 4.0%. In gender comparison, we believe that there are differences between male and female NPD, male frequency is higher than female frequency, which is consistent with Torgersen’s research [21]. Furthermore, NPD patients characterized as having a higher education level than others, suggesting that NPD patients might be used to be high function. These exploratory data will help Chinese psychiatrists reconsider the applicability of NPD in the Chinese population.

The co-morbidity with other types of PDs is also one of the main problems hindering the application of NPD in China. In this study, we found that NPD likely overlaps with Histrionic PD and Borderline PD, which are both in cluster B, whereas Paranoid PD is in cluster A. These three types of PD also entered the logistic regression model at the same time, indicating that patients with any of these three PDs are more likely to meet NPD criteria as well. Clinical discrimination is needed to compare NPD with these types of PDs.

From 1914, when Freud published his dissertation, “On Narcissism,” to 1931, when Freud formally proposed narcissistic personality types, NPD diagnosis has been growing. With the continuous development of narcissism theory since 1980, NPD diagnostic criteria have entered continuous revision in the DSM diagnostic system, and the whole process has spanned almost a century. The current diagnostic criteria of NPD in the DSM-IV can be clearly compared with the symptoms of patients in clinical application, and many experimental studies have proved that NPD can be used as an independent diagnostic criterion [22, 23]. However, this historical background of the completely western culture-based diagnosis criteria of NPD may cause overlap and be confused with other types of PDs when NPD applied in countries with different cultural backgrounds [24]. Future research should attempt to answer the question as to what these cultural differences are. This then leads to the question as to how to modify the NPD criteria to fit in a Chinese cultural context.

Consistently, all criteria for NPD in the DSM-IV were arranged in descending order of importance [25]. This order has also been confirmed to some extent in this study. The order of the positive rate of NPD criteria is 1, 2, 4, 6, 5, 3, 8, 7, 9 (criterion number). However, we also found those NPD diagnostic criteria are common in other types of PDs in Cluster B. This is also confirmed by Morey et al. [26]. The co-morbidity rate of NPD and Histrionic PD is as high as 53.1% followed by Borderline PD (46.9%). It has been suggested that the clinical differentiation of other types of Cluster B PDs should be strengthened when NPD diagnostic criteria are applied in China. The current NPD diagnosis is a categorical approach which has been questioned for its arbitrary diagnostic thresholds and extensive overlap with other PD categories in DSM-IV. The current global trend is that the categorical PD diagnoses transfer to the dimensional-categorical (DSM-5) and dimensional (ICD-11) approaches. Further studies should also examine whether dimensional approaches can improve the validation of NPD diagnosis in Chinese population.

Our study has several limitations, including that the study was designed as a cross-sectional, open and single-center study. The data may not be representative of the entire Chinese population since recruitment were conducted only at a single site. However, the SMHC is the largest psychiatric service center in China (serving over 800,000 outpatients per year) and provides professional treatment for patients throughout the country, and about half of the current sample were not Shanghai natives. However, although a single-site design may increase sample homogeneity and continuity, it also could limit the generalisability of the findings. The SCID-II clinical interview was only conducted for those whose self-reported PDQ-4+ screening was positive that could result in underestimates of the frequency of NPD by the exclusion of participants who were screened negative. Finally, our sample is recruited from outpatients, the possibility of clinical state effects may impact our PD assessment.

## Conclusions

In summary, although the diagnosis of DSM-5 NPD is not included in Chinese psychiatric practice, our results suggest that NPD is not rare in psychiatric outpatients in China. Further longitudinal studies should examine the validity and reliability of NPD diagnosis among Chinese patients using a prospective design.

## Data Availability

The dataset(s) generated during the current study are not publically available due to ethical restrictions but are available from the corresponding author on reasonable request.

## Abbreviations

CCMD-3: Chinese Classification and Diagnosis of Mental Diseases, Third Edition
CI: confidence interval
DSM-III: Diagnostic and Statistical Manual of Mental Disorders, third edition
ICD-11: The 11th revision of the International Classifications of Diseases and Related Health Problems
NPD: Narcissistic Personality Disorder
OR: Odds ratios
PDQ-4 +: Personality Diagnostic Questionnaire Fourth Edition Plus
SCID-II: Structured Clinical Interview for DSM-IV Axis II
